# Cytomegalovirus and disseminated histoplasmosis-related hemophagocytic lymphohistiocytosis syndrome in an HIV-patient late presenter with IRIS: a case report

**DOI:** 10.1186/s12981-020-00304-0

**Published:** 2020-08-14

**Authors:** Luz A. González-Hernández, Monserrat Alvarez-Zavala, Rodolfo I. Cabrera-Silva, Pedro Martínez-Ayala, Fernando Amador-Lara, Aída S. Ramírez-González, Ana L. Ron-Magaña, Vida V. Ruiz Herrera, Karina Sánchez-Reyes, Jaime F. Andrade-Villanueva

**Affiliations:** 1 HIV Unit, Antiguo Hospital Civil de Guadalajara “Fray Antonio Alcalde”, Guadalajara, Jalisco Mexico; 2 HIV and Immunodeficiencies Research Institute, CUCS-University of Guadalajara, Guadalajara, Jalisco Mexico; 3 Hematology and Trasplant Unit, Hospital Civil de Guadalajara “Fray Antonio Alcalde”, Guadalajara, Jalisco Mexico

**Keywords:** Hemophagocytic lymphohistiocytosis syndrome, Cytomegalovirus, Disseminated histoplasmosis, HIV, Applied translational medicine, IRIS, Case report

## Abstract

**Background:**

Hemophagocytic lymphohistiocytosis syndrome (HLS) is an immune-mediated life-threatening disease considered as a medical emergency, with a potentially fatal multisystem inflammatory outcome. We present a patient that developed HLS and was able to be diagnosed efficiently with the help of an academic research institute of immunology.

**Case presentation:**

A 21 years old male Mexican with human immunodeficiency virus (HIV), late presenter; who developed cytomegalovirus (CMV) infection and a disseminated histoplasmosis-related HLS, as part of an immune reconstitution inflammatory syndrome (IRIS). The patient required a long course of corticotherapy, intravenous immunoglobulin and massive transfusions (more than 10 units in 24 h, and a total of 83 units), besides amphotericin-B and ganciclovir treatment. An academic research institute of immunology aided in the accurate diagnosis of HLS with the implementation of tests not available within the hospital, thus improving the care provided to the patient. The patient recovered, was discharged, and continue to improve.

**Conclusion:**

The objective of this report is to highlight the importance of having multidisciplinary support, including basic medical sciences groups providing specific tests that are sometimes very difficult to get, which provides a benefit to patients in the well-aimed diagnosis as part of applied translational medicine.

## Background

HLS is an immune-mediated life-threatening disease considered as a medical emergency, with a potentially fatal multisystem inflammatory outcome, mainly if the correct treatment is not applied early [1, 2]. Antiretroviral treatment (ART) initiation is sometimes associated with a generalized inflammatory response termed IRIS, which occurs in two forms: (1) “unmasking” IRIS refers to the flare-up of an underlying, previously undiagnosed infection soon after ART is started and (2) “paradoxical” IRIS refers to the worsening of a previously treated disease after ART initiation [3, 4]. We report here a young Mexican man with HIV-infection and an unmasking IRIS; who came to receive medical attention for oral ulcers, fever, diarrhea, myalgias, dyspnea, and pancytopenia. His thrombocytopenia worsened, and he developed profuse hemorrhage (up to 1.5 L/day), requiring massive blood transfusions, intravenous immunoglobulin, and corticotherapy. The HLS diagnosis was made with the support of an academic research institute of immunology focused on HIV and Immunodeficiencies (HIV and Immunodeficiencies Research Institute; InIVIH). The overall goal of InIVIH is to investigate the main molecular and immunological mechanisms involved in the pathogenesis of HIV and its complications in Mexican patients. With the expertise of the researchers doing specialized techniques, an accurate diagnosis was achieved and allowed for prompt treatment for the patient. This collaboration highlights the importance of having an applied translational medicine, especially in places where up to 79% of the HIV-infected patients are late presenters (patients starting antiretroviral therapy for HIV infection with CD4^+^ T cells less than 200 cells/μL), and specialized techniques are unavailable or unaffordable [5].

## Case Presentation

A 21-year-old male Mexican with HIV infection with a basal HIV viral load of 70,800 copies/mL, CD4^+^ T cells of 6 cells/μL (3%) and CD8^+^ T cells of 88 cells/μL (46%), who started ART (abacavir/lamivudine/dolutegravir) one-month prior symptoms, came to us with 12 days of clinical evolution and complaining about a lower lip ulceration, fever, chills, diarrhea (Bristol Stool Scale: type 5), myalgias, progressive dyspnea, and generalized weakness. The patient had an arterial pressure of 98/60 mmHg, fever (38 °C), cachexia, dehydration, tachycardia (140 beats/min) without murmurs, polypnea (35 breaths/min), and multiple tattoos. Right eye with phthisis bulbi (due to previous ocular trauma), with painful and erythematosus lower lip ulcerations and painful cervical adenopathies (0.5–2.5 cm in diameter). Pulmonary examination reported a bilateral decrease of the vesicular murmur and oxygen saturation of 85% by pulse oximeter. The patient presented hepatosplenomegaly, diffuse abdominal pain, and genital warts; neuromuscular alterations were not detected.

In the emergency department, he was diagnosed with sepsis, acute kidney injury KDIGO III and pancytopenia: hemoglobin: 6.0 g/dL (12.2–18.1 g/dL), white blood cell count: 2.15 × 10^3^/μL (4.6–10.2 × 10^3^/μL), and platelet count: 21.53 × 10^3^/μL (142–424 × 10^3^/μL). Moreover, the patient had lactate: 4.3 mmol/L, procalcitonin: 0.43 ng/mL, triglycerides: 284 mg/dL (3.20 mmol/L), D-Dimer 4557 ng/mL, C-reactive protein: 56.9 mg/L, creatinine: 1.38 mg/dL (0.5–1.2 mg/dL), urea: 92.9 mg/dL (15–39 mg/dL), albumin: 1.3 g/dL, aspartate aminotransferase (AST): 173 IU/L, lactate dehydrogenase (LDH): 1059 IU/L, fibrinogen: 233 mg/dL (2.3 g/L), prothrombin time: 15.1/s, and Hepatitis C virus-positive (Child-Turcotte-Pugh Class B [Score 7]) and APRI Index 0.016 (Table 1). Urinalysis without pathological findings.

**Table 1:** Relevant laboratory tests from baseline to discharge

Test (units) (reference values)	Day 0	Day 6	Day 15	Day 23	Day 29	Day 62
Hemoglobin (g/dL) (12.2–18.1)	6.0^d^	8.04	6.3	7.9	12.2	11.01
Platelets (× 10^3^/μL) (142–424)	21.53^d^	6.99	39.75	30.98	350.10	112.40
White blood cells (× 10^3^/μL) (4.6–10.2)	2.15^d^	2.13	10.68	3.00	4.54	2.09
Neutrophils (× 10^3^/μL) (37–80%)	1.72	1.97	10.03	2.75	4.29	1.58
Creatinine (mg/dL) (0.5–1.2)	1.38	1.4	0.92	0.36	0.87	0.41
Urea (mg/dL) (15–39)	92.9	90	103.9	44	25.8	26
Glucose (mg/dL) (60–125)	125^a^	159^b^	116^b^	130^b^	101	86
Sodium (mEq/L) (135–145)	126	128	128	134	138	137
Potassium (mEq/L) (3.5–5.1)	5.6	5.4	5.0	3.73	3.6	3.47
ALT (IU/L) (10–40)	20	33	25	19	13	9
AST (IU/L) (10–50)	173	83	32	18	18	9
CK (U/L) (38–174)	40	–	–	–	–	–
Albumin (g/dL) (3.5–5.0)	1.3	1.97	2.03	2.00	2.14	3.82
Total Bilirubin (mg/dL) (0–1)	1	0.5	0.2	0.25	0.6	0.31
LDH (IU/L) (91–190)	1059	208	127	100	116	100
PT (s) (9.5–13)	15.1	14.3	16	14	15.4	11.5
PTT (s) (25.5–35.5)	43.2	42.2	39.2	49	39.1	34.7
INR [1]	1.36	1.29	1.54	1.35	1.39	1.10
Triglycerides (mg/dL) (35–160)	284^d^	–	–	–	–	84
Fibrinogen (mg/dL) (200–480)	233^d^	553	683	864	633	613
Ferritin^c^ (ng/mL) (20–250)	> 5000^d^	–	4061.7	–	–	–
D-Dimer (ng/mL) (< 500)	4557	–	–	1929	–	–
CRP (mg/L) (< 10)	56.9	–	–	12.7	–	–

PT, Prothrombin time; TPT, partial thromboplastin time; INR, international normalized ratio; ALT, alanine aminotransferase; AST, aspartate aminotransferase; LDH, lactate dehydrogenase; CRP, C-reactive protein; CK, creatine kinase

^a^Non-fasting glucose; ^b^ patient under corticotherapy; ^c^ The ferritin lab test provided in our hospital has an upper max limit of 5000 ng/mL; ^d^ Parameter that fulfilled HLS diagnostic criteria

**Fig. 1:**
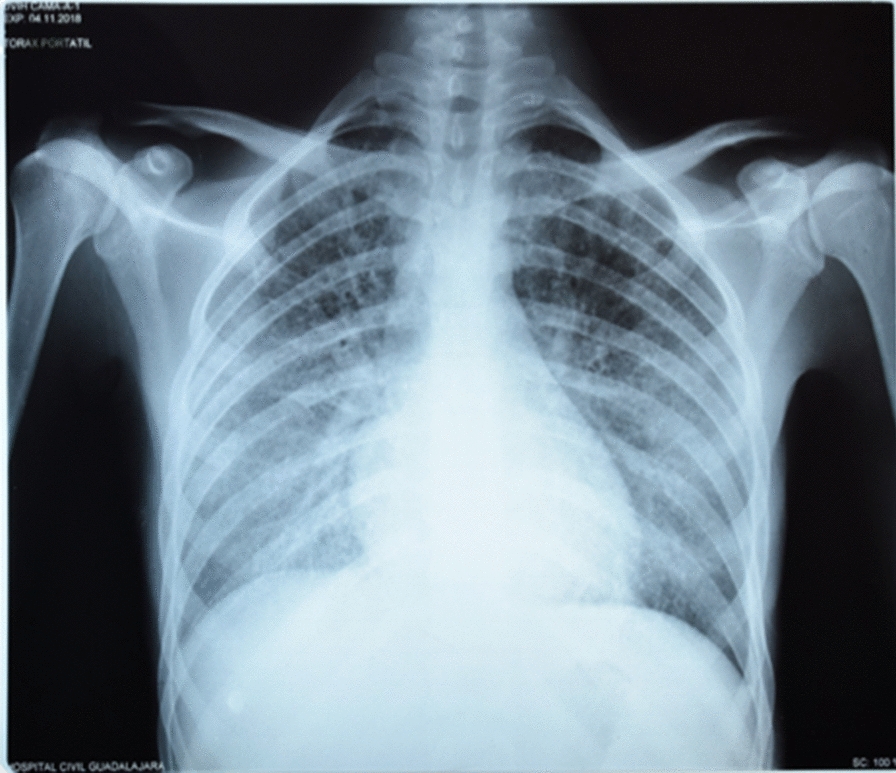
Chest X-ray shows fine bilateral reticulo-interstitial infiltrates

**Fig. 2:**
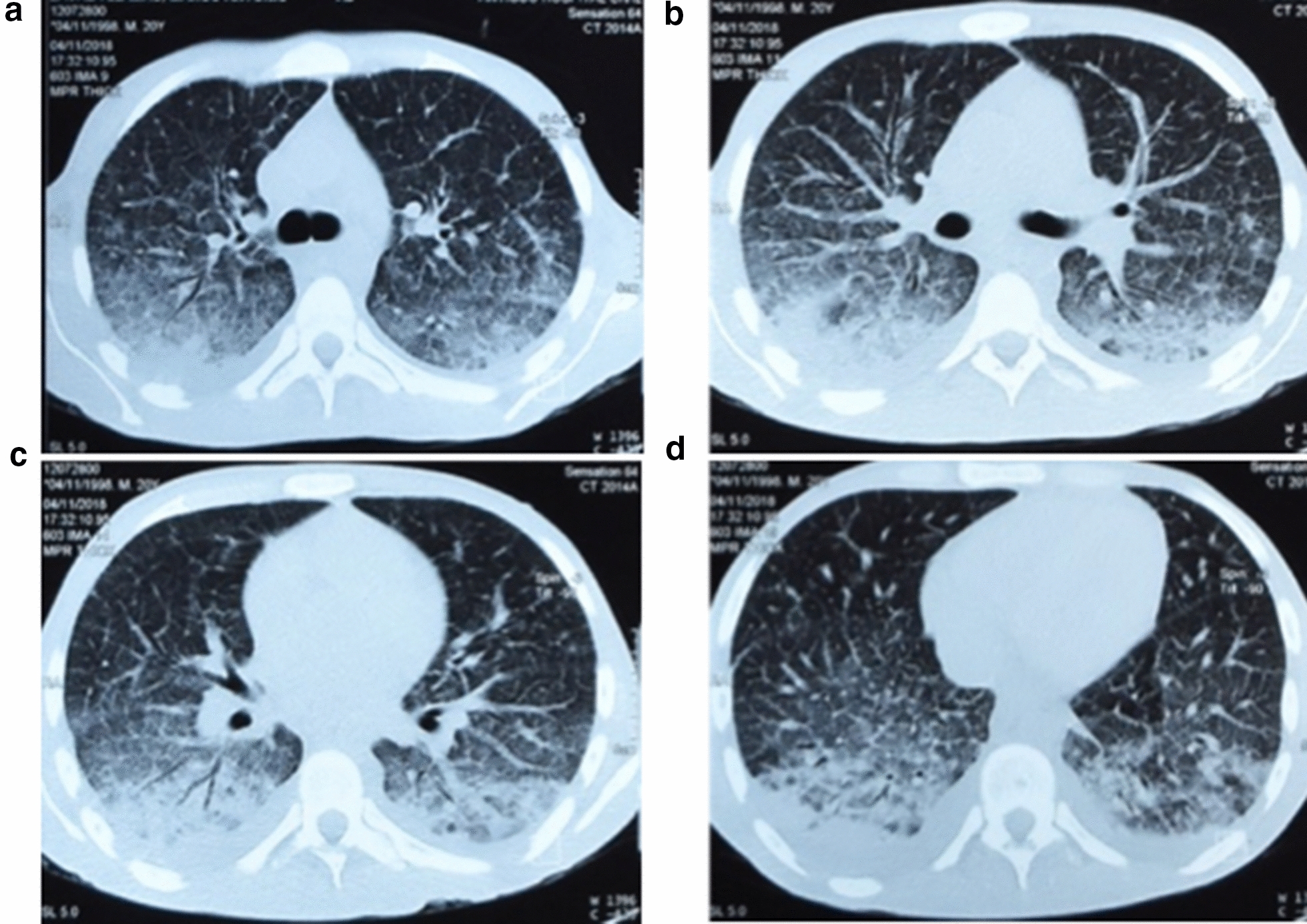
**a**–**d** A chest computed tomography shows bilateral basal consolidation areas, interstitial and reticulonodular infiltrates at different levels

A chest X-ray showed fine bilateral reticular interstitial infiltrates (Fig. 1), and a computed tomography scan showed bilateral basal consolidation areas, interstitial and reticulonodular infiltrates (Fig. 2), suggestive of *Pneumocystis jiroveci* pneumonia (PCP).

**Fig. 3:**
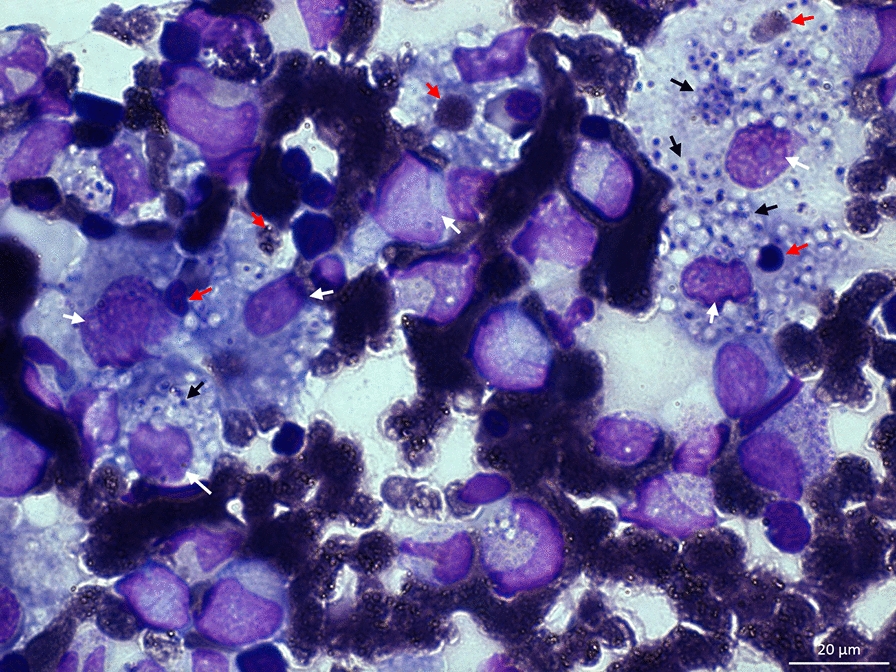
Bone marrow aspirate shows reticulohistiocytic hyperplasia (white arrows) with phagocytized erythrocytes (red arrows), platelets, and abundant *Histoplasma capsulatum* yeast cells inside the cytoplasm (black arrows). X100 optical zoom with oil immersion

**Fig. 4:**
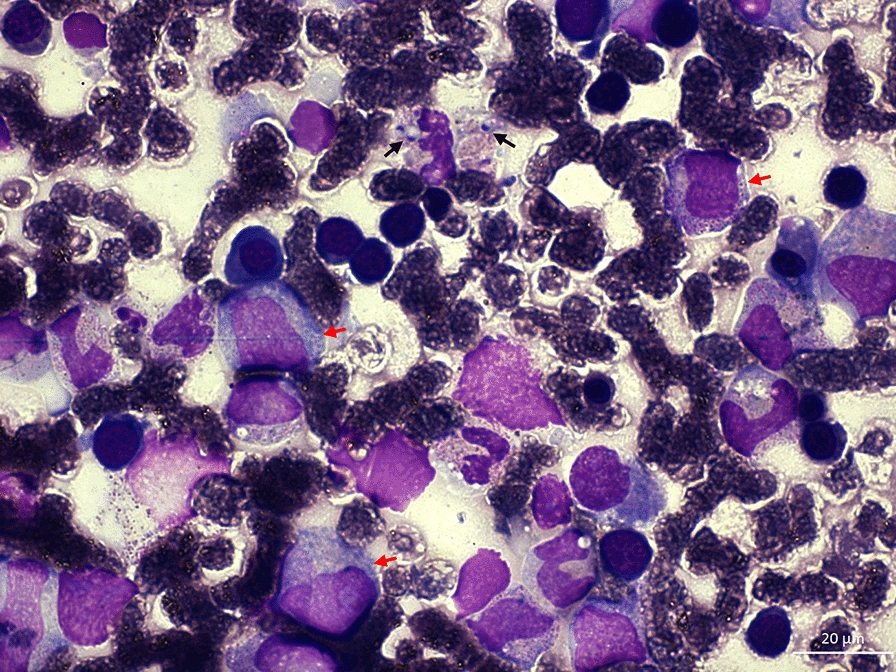
Bone marrow aspirate shows monocytic hyperplasia (red arrows) with the presence of *Histoplasma capsulatum* spherules disperse through-out the aspirate (black arrows). X100 optical zoom with oil immersion

Secondary to the fever, pancytopenia, and > 1000 IU/L LDH at day one, 3 mg/kg/day of liposomal amphotericin B was given empirically to treat probable Histoplasmosis and, due to suggestive radiological findings, empirical 160 mg/800 mg trimethoprim/sulfamethoxazole (TMP/SMX) was administered for PCP; as well as transfusions of fresh frozen plasma, platelets, and red blood cells. The fever improved; however, the hemorrhage and pancytopenia worsened, the lowest platelet count detected on the 4th day of hospital stay (Table 1). Therefore, we performed a bone marrow aspiration (BMA), where lymphoma and malignancy were discarded. Through a Wright-Giemsa staining, we observed *Histoplasma capsulatum*, an increased number of macrophages with active hemophagocytosis (Fig. 3, 4), and with a probable diagnosis of disseminated intravascular coagulation (DIC; a score of 6 according to the consensus guideline by hematologists as part of the International Society of Thrombosis and Hemostasis [ISTH] [6]). With the support of InIVIH, we evaluated the natural killer (NK) cells cytotoxic activity. By assessing membrane expression of CD107a in NK cells using flow cytometry after cytotoxic assays, we confirmed a significant reduction of the NK cells cytotoxicity. Sadly, due to the lack of reagents, the measurement of soluble α-chain interleukin 2 receptor (sCD25) could not be performed.

The diagnosis of HLS was made based on (I) fever, (II) hepatosplenomegaly, (III) pancytopenia, (IV) hemophagocytosis in bone marrow aspirate smear, (V) hypertriglyceridemia, (VI) hypofibrinogenemia, (VII) hyperferritinemia (> 500 ng/mL) and (VIII) defective NK cells degranulation and cytotoxicity (H-score of:276, with 99.8% chance for HLS); thus, we started a daily dose of prednisone 40 mg. The patient had an inadequate response, and after 25-days of corticotherapy, he presented severe epistaxis refractory to platelets transfusion (51 units), romiplostim, and ethamsylate, which required nasal tamponade. He also developed a profuse hematochezia (up to 1.5 L/day), that required massive transfusions (more than ten units of packed red blood cells in 24 h and nine units of fresh frozen plasma) and tranexamic acid. This clinical evolution supported the empirical initiation of ganciclovir therapy, in addition to 3 days of intravenous immunoglobulin, while waiting for the CMV viral load results. The pancytopenia progressively improved, and the CMV qPCR test reported 237,616 IU/mL, confirming CMV infection.

**Fig. 5:**
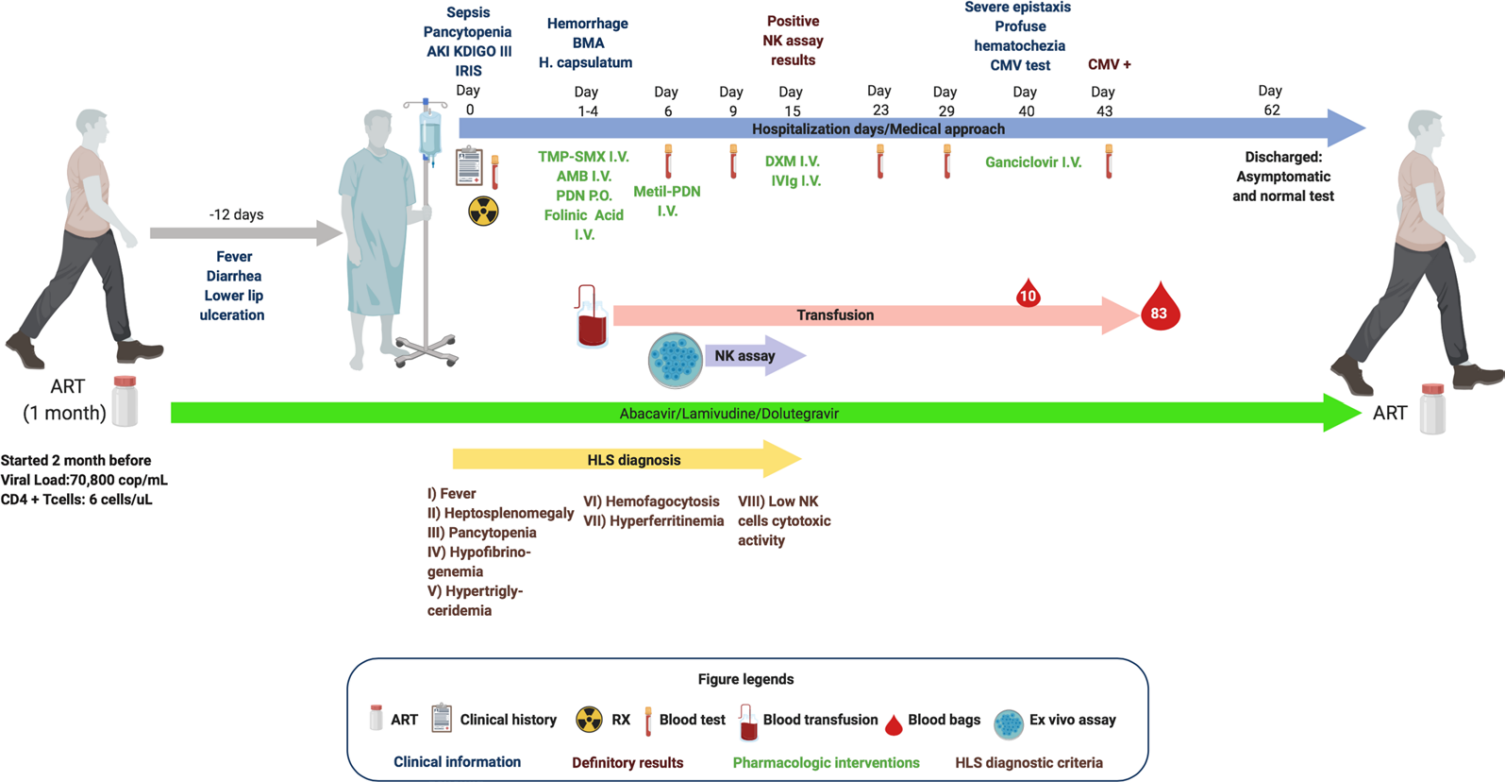
After ART initiation, the patient began with fever, chills, lower lip ulceration, and diarrhea 12 days prior to hospitalization (gray arrow). On day 0, the patient was diagnosed with sepsis, pancytopenia, AKI (KDIGO III), and IRIS; blood tests, Chest X-ray, and CT scan studies were performed. On day 1, empirical treatment for severe PCP and disseminated histoplasmosis was initiated; also, the patient started with hemorrhage, and blood transfusions (platelets, plasmapheresis, and red blood cells) were administered, which continued intermittently until day 43 (red arrow). On day 2, hemophagocytosis and *H. capsulatum* were reported in a BMA. On day 9, support was requested to InIVIH, and samples were collected for NK cells cytotoxic assays. On day 13, low NK cells cytotoxic activity was confirmed (purple arrow), and a 3-day immunoglobulin treatment was provided. On day 40, severe epistaxis and hematochezia required massive transfusions (10 units in less than 24 h; indicated by a blood droplet); empirical ganciclovir was provided while test results were reported. On day 43, CMV infection was confirmed. After 62 days and 83 blood transfusions, the patient was discharged with undetectable HIV viral load, 10 CD4^+^ T cells count, and normal blood tests (blue arrow). During all this time, ART treatment was not suspended nor modified (green arrow). The yellow arrow indicates the process of HLS diagnosis. Created with BioRender. AKI: Acute Kidney Injury; AMB: Amphotericin B; ART: Antiretroviral Therapy; BMA: Bone Marrow Aspiration; CMV: Citomegalovirus; CT: Computed Tomography; DXM: Dexametasone; HLS: Hemophagocytic Lymphohistiocytosisi Syndrome; IRIS: Inmmune Reconsitution Inflammatory Syndrome; I.V: Intravenous; IVIg: intravenous gamma Immunoglobulin; Methyl-PDN: Methylprednisolone; PCP: *Pneumocystis jiroveci* Pneumonia; PDN: prednisone; P.O: oral administration; TMP-SMX: trimethoprim/sulfamethoxazole

The patient completed his treatment for disseminated histoplasmosis with amphotericin B for 14 days, followed by itraconazole as maintenance management. He was discharged 62 days after admission, with undetectable HIV viral load (< 40 copies/mL), CD4^+^ T cells of 10 cells/μL (10%), and normalized standard laboratory studies. He remained stable in the subsequent clinical follow-up (Table 1). A summary of the patient’s clinical evolution is shown in Fig. 5.

## Discussion and conclusions

Described in 1939 by Scott and Robb-Smith, HLS is an immune-mediated life-threatening disease, which is characterized by uncontrolled hyperactivation of macrophages, histiocytes, and an impaired NK cells and cytotoxic T cell function, with a potentially fatal multisystem inflammatory outcome [1, 2]. HLS could be primary (or genetic, mainly reported in children, and discarded in this case) and secondary (or reactive). HLS is associated with many medical conditions, such as viral, bacterial, parasitic, and fungal infections, neoplasms, autoimmune diseases, transplants, surgery, medications, vaccination, diabetes mellitus, chronic liver disease, pregnancy, and hemodialysis; sometimes, HLS remained idiopathic or with unknown etiology [2, 7].

In adults, the male–female ratio is 1:7, and the age at diagnosis is around 50 years. The principal factors that trigger HLS are external (drugs and infections) and, frequently, there is a basal underlying disease or disorder predisposing it (e.g., hematological cancer, systemic lupus erythematosus, or HIV infection) [2, 8].

Within HLS’s pathophysiology, a low or absent NK cell activity occurs by a defect in granule-mediated cytotoxicity. Secondary to the absence of perforin and FAS systems (part of the inflammation control), there are an enhanced antigen presentation and uncontrolled activation of macrophages, histiocytes, and T cells. These cells create a cytokine storm, which develops a hemophagocytic lymphohistiocytosis, contributing to tissue injury, progressive systemic organ failure, and death [9].

Also, the release of pro-inflammatory cytokines promotes the hemophagocytosis, a hallmark of HLS [7]. Previous reports indicate that the presence of hemophagocytosis has a sensitivity of 80–83% and a specificity of 60% for HLS; nevertheless, hemophagocytosis can be absent in some cases [10].

Often, the differential diagnosis between HLS, DIC, and sepsis is a challenge and, sometimes, it may be even impossible to rule out the coexistence of these pathologies. In a febrile patient without a transfusion history, no defect in the iron metabolism, pronounced cytopenias, and the presence of splenomegaly and high hyperferritinemia (> 2000 ng/mL), should guide the clinician towards the search for HLS [11]. Since the beginning, this case fulfilled six of the 8 criteria of the HLS-2004 guidelines (Table 2). These findings encouraged us to look for a defect in NK cell’s cytotoxic activity to complete the diagnosis criteria. Regarding DIC, it is important to mention that the patient maintained a score of 6 (probable overt DIC) throughout his hospitalization until day 23 when his score decreased to 3 (not overt DIC). The DIC score was developed as a consensus guideline by hematologists as part of the ISTH. It is designed to be applied to patients with a critical illness known that could precipitate DIC and categorizes patients into “probable overt DIC” and “not overt DIC” based on a series of laboratory parameters. This score is instrumental in tracing suspicion of DIC, and it is recommended to repeat the score every 1 or 2 days [6].

**Table 2:** Diagnosis Guidelines for HLS

Diagnostic Guidelines for HLS
1. Molecular diagnosis consistent with HLS
Mutations of PRF1, UNC13D, STXBP1, RAB27A, STX11, SH2D1A or XIAP
OR
2. Five out of the eight following criteria
Fever (> 38.4)
Hepatomegaly and/or Splenomegaly
Cytopenia (Hemoglobin < 9.0 g/dL; and/or Platelets < 100 per 10^9^/L; and/or neutrophil < 1 cell per 10^9^/L)
Hypertriglyceridemia (> 265.5 mg/dL while fasting) and/or Hypofibrinogenemia (< 274 mg/dL)
Ferritin > 500 ng/mL
Hemophagocytosis in bone marrow, spleen, lymph node, or liver
Low or absent NK cells cytotoxic activity
Elevated soluble α-chain interleukin 2 receptor (≥ 2400 IU/mL)

Following ART initiation, HIV+ patients can have an exaggerated inflammatory reaction to pathogenic microorganisms when the immune system begins to recover [12], a condition that could worsen the HLS. These events are known as IRIS (it can be paradoxical or unmasking), which have been reported most commonly in HIV late presenters with severe immunosuppression (≤ 100 cells/μL of CD4^+^ T cells) [4]. Since there is no diagnostic test for IRIS, confirmation of the syndrome relies heavily on clinical and laboratory data; however, there is not a standardized definition for diagnosis. Shelburne and et al. describe IRIS on HIV infection with effective ART (i.e., a decrease in HIV-1 RNA concentration or an increase CD4^+^ T cells count from baseline), and with clinical symptoms consistent with an inflammatory process. Besides, the clinical course should not be compatible with the expected course for previously diagnosed opportunistic infection, a newly diagnosed opportunistic infection, or drug toxicity [12, 13].

IRIS manifestations concord with the type of opportunistic infection, the latter depending on the epidemiology of each region. In Mexico, the leading causes reported are *Mycobacterium tuberculosis* infection, disseminated *Mycobacterium avium* complex, *Pneumocystis jirovecii*, Cryptococcosis, Histoplasmosis, CMV, and varicella-zoster virus. IRIS can be mild or life-threatening, as there is around two times more risk of death among patients with IRIS compared with patients without it [4, 14].

CMV infects the majority of adults, and during the primary infection, it can course with transient neutropenia and thrombocytopenia, followed by an asymptomatic viral latency period. CMV reactivation is more common in immunocompromised patients with depressed cell-mediated immunity, such as in HIV-infection. There are reports about pancytopenia secondary to CMV infection or reactivation [15, 16]. Several mechanisms have been proposed to explain the deleterious effect of CMV on human hematopoietic function, including (1) Alteration of cell function by the production of inhibitory cytokines, (2) Perturbation of stromal cell functions with the subsequent reduced production of hematopoietic factors or disturbed expression of cell surface adhesion molecules, and (3) The direct infection of the hematopoietic stem or progenitor cells [17, 18]. CMV infection in the bone marrow is a rare condition and is suspected when there are atypical cells in a BMA. A streptavidin–biotin–peroxidase detection system and diaminobenzidine staining can be used to identify a CMV bone marrow infection, as well as immunohistochemistry [19]. In this case report, there were no atypical cells in the BMA; therefore, immunohistochemistry was not performed. The pathogenesis of pancytopenia secondary to CMV infection is multifactorial; consequently, a broad diagnostic approach is required. Furthermore, AIDS patients sometimes require other drugs that could impact the bone marrow function, such as the case of TMP/SMX [17, 18], which in this case was given as treatment due to suspicion of PCP. However, cytopenia began prior to the administration of TMP/SMX, ruling out this possibility. Also, 15 mg of folinic acid was administered daily since the pancytopenia was detected.

In patients with HIV infection, HLS could initiate by opportunistic or non-opportunistic diseases, neoplasms, the HIV itself, or even by ART initiation, especially in late presenters, mainly in patients who developed IRIS [20]. The literature suggests that the host genetic factors, rather than viral pathogenesis, determine the occurrence of HLS [21].

The clinical features of HLS are diverse and nonspecific; moreover, other inflammatory conditions can cause them. Thus, to assess the diagnosis of HLS, it is imperative to consider the updated criteria proposed by ‘The Histiocyte Society’ in 1991 (HLS 2004 guideline). The guidelines diagnose HLS by the molecular alterations consistent with HLS (for primary HLS), or by getting five out of eight criteria shown in Table 2 (for reactive HLS). The HLS 2004 guidelines have been found to have a similar specificity and sensibility to the H-score. For the HLS 2004 guidelines, the sensitivity and specificity with at least 5 criteria is 80% and 95%, respectively, while and H-score with > 185 points has 85% and 88%, respectively [22].

Nonetheless, the HLS guidelines’ criteria referred above are not available in many care centers, including our hospital, as many of the techniques required are not available, or they are unaffordable for the hospital or the patient. However, thanks to the support from InIVIH, we could evaluate the NK cells’ cytotoxicity; we evaluated NK cells cytotoxic activity by flow cytometry. In isolated and stimulated NK cells, we measured the membrane expression of CD107a, a marker of immune cell activation, and cytotoxic degranulation for NK cells and CD8^+^ T cells [23–25]. Through this assay, we detected the low expression of NK cells (0.6–5.1%; normal expression goes around 10.3–17.4%), thus fulfilling the criteria.

As mentioned previously, sCD25 levels were not measured due to the lack of adequate reagents. However, an indirect evaluation for sCD25 was attempted. By flow cytometry, concentrations of membrane CD25 on CD8^+^ T cells were measured and found a 30% increase of CD25 levels when compared to a healthy person (age and sex paired). As the patient had low CD4^+^ T cell count, we hypothesized that the primary source of sCD25 is from the CD8^+^ T cell. As CD8^+^ T cells had an increased amount of membrane CD25, they could, eventually, cause high levels of sCD25; hence filling the last criteria for HLS.

Regardless, as this is not a confirmed technique for the measurement of sCD25, we did not add this measurement in the scoring and diagnosis of HLS in this patient.

Recently, the introduction of the H-Score redefined the cutoffs of the Histiocyte Society’s HLS diagnosis; thus, adding the (I) underlying immunosuppression, (II) hepatosplenomegaly and (III) AST > 30 IU/L to their criteria. Currently, the H-Score is mainly validated for reactive HLS. The probability of having HLS ranged from < 1% with an H-Score of 90 points or less to > 99% with an H-Score of 250 [26, 27]. The patient in this case report had a 276 H-Score.

Reactive HLS carries a high mortality, ranging from 52 to 74% at 1 year, depending of variables like patients older than 30 years, the presence of disseminated intravascular coagulation, elevated ferritin level, and anemia with accompanying thrombocytopenia [28]. HLS treatment consists of dexamethasone and etoposide [10]. In this paper, we described a patient with acquired immunodeficiency syndrome who presented HLS related to an unmasked IRIS, secondary to disseminated histoplasmosis and CMV infection. The patient required more than 80 transfusion units, romiplostim, tranexamic acid, a long course of corticotherapy (without etoposide), intravenous immunoglobulin, and a long list of supportive measures. Although the patient was in a critical condition, the early identification of HLS allowed the implementation of the right treatment. The aid provided by InIVIH hastened the diagnosis and led to prompt therapy. Also, it highlighted the importance of having an applied translational medicine with medical sciences research groups, especially in places where there are many HIV late presenters with the risk of developing pathologies with a difficult diagnosis.

HLS is a multifaceted, potentially life-threatening clinical syndrome without specific clinical findings; thus, HLS requires a high degree of suspicion and a multidisciplinary approach by clinicians (including immunologists, hematologists, and infectious disease specialists) for the early diagnosis and the correct treatment of the underlying etiology, plus an immunosuppressant therapy. For HLS, some diagnostic clues include high fever (unresponsive to broad-spectrum antibiotics), hypertriglyceridemia, hypofibrinogenemia, hyperferritinemia, cytopenia, visceromegaly, and a BMA smear with hemophagocytosis. Furthermore, having access to a research institute that provides support with a specialized immunological assay, not only makes it easier for clinicians to have greater certainty in the diagnosis of HLS but it can also accelerate it, mainly in places where these types of lab test are not available or affordable -like in our settings. Therefore, we want to emphasize the need for collaboration with research laboratories in the clinical field to offer more accurate diagnoses through these specialized tests, which are sometimes part of the diagnostic criteria, like in HLS. This case report shows that the collaboration between medical and basic sciences is essential for the progression of translational medicine and the improvement of medical care. It is clear that the early identification of the causative agents, made possible thanks to the support of a research group, as well as the initiation of the appropriate treatment, significantly contributed to this patient’s survival.

## Data Availability

All data generated or analyzed during this study are included in this published article.

## Abbreviations

AIDS: Acquired Immunodeficiency Syndrome
ART: Antiretroviral Treatment
AST: Aspartate Aminotransferase
BMA: Bone Marrow Aspiration
CD25: Interleukin 2 Receptor
sCD25: Soluble Interleukin 2 Receptor
CMV: Cytomegalovirus
DIC: Disseminated Intravascular Coagulation
HIV: Human Immunodeficiency Virus
HLS: Hemophagocytic Lymphohistiocytosis Syndrome
IRIS: Immune Reconstitution Inflammatory Syndrome
ISTH: International Society of Thrombosis and Hemostasis
InIVIH: HIV and Immunodeficiencies Research Institute
LDH: Lactate Dehydrogenase
NK cells: Natural Killer cells
PCP: *Pneumocystis jiroveci* Pneumonia
TMP/SMX: Trimethoprim/Sulfamethoxazole
